# The roles of ubiquitin–proteasome system and regulator of G protein signaling 4 in behavioral sensitization induced by a single morphine exposure

**DOI:** 10.1002/brb3.2922

**Published:** 2023-02-15

**Authors:** Yanting Wang, Xingzi Hou, Shoupeng Wei, Jiaqing Yan, Zhe Chen, Mingyu Zhang, Qingying Zhang, Yingyuan Lu, Qingjie Zhang, Tiange Zheng, Jingyi Jia, Bin Dong, Ying Li, Yuanyuan Zhang, Jianhui Liang, Guohui Li

**Affiliations:** ^1^ Department of Pharmacy, National Cancer Center/National Clinical Research Center for Cancer/Cancer Hospital Chinese Academy of Medical Sciences and Peking Union Medical College Beijing China; ^2^ State Key Laboratory of Natural and Biomimetic Drugs and Department of Natural Medicines, School of Pharmaceutical Sciences Peking University Health Science Center Beijing China; ^3^ Laboratory for Integrative Neuroscience, National Institute on Alcohol Abuse and Alcoholism National Institutes of Health Bethesda Maryland USA; ^4^ Department of Molecular and Cellular Pharmacology, School of Pharmaceutical Sciences Peking University Beijing China

**Keywords:** behavioral sensitization, morphine, opioid addiction, regulator of g protein signaling 4, ubiquitin–proteasome system

## Abstract

**Aims:**

Opioid addiction is a major public health issue, yet its underlying mechanism is still unknown. The aim of this study was to explore the roles of ubiquitin–proteasome system (UPS) and regulator of G protein signaling 4 (RGS4) in morphine‐induced behavioral sensitization, a well‐recognized animal model of opioid addiction.

**Methods:**

We explored the characteristics of RGS4 protein expression and polyubiquitination in the development of behavioral sensitization induced by a single morphine exposure in rats, and the effect of a selective proteasome inhibitor, lactacystin (LAC), on behavioral sensitization.

**Results:**

Polyubiquitination expression was increased in time‐dependent and dose‐related fashions during the development of behavioral sensitization, while RGS4 protein expression was not significantly changed during this phase. Stereotaxic administration of LAC into nucleus accumbens (NAc) core inhibited the establishment of behavioral sensitization.

**Conclusion:**

UPS in NAc core is positively involved in behavioral sensitization induced by a single morphine exposure in rats. Polyubiquitination was observed during the development phase of behavioral sensitization, while RGS4 protein expression was not significantly changed, indicating that other members of RGS family might be substrate proteins in UPS‐mediated behavioral sensitization.

## Graphical abstract

(1) ubiquitin‐proteasome system positively participates in the establishment of behavioral sensitization; (2) the expression of regulator of G protein signaling 4 was not changed during the development of behavioral sensitization; 3) the roles of other family members of regulators of G protein signaling in opioid addiction and their relations with UPS are worth being further explored

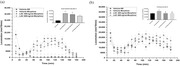

## 1 INTRODUCTION

Opioid abuse and addiction are not only medical problems that limit the clinical application of opioids but they also cause accidental death and many social problems (Damiescu et al., 2021; Ehrlich et al., 2019; United Nations Office on Drugs & Crime, 2020). Currently, methadone, buprenorphine, and naltrexone are mainly used to treat opioid addiction, but the existing treatment methods generally have shortcomings such as poor medication compliance and a high rate of relapse (Volkow et al., 2019). The critical point in treatment of addiction is to prevent craving and relapse, of which the molecular mechanisms have not been clear (Kakko et al., 2019). Therefore, in‐depth exploration of the molecular mechanisms underlying opioid addiction has great medical and social significance.

Ubiquitin–proteasome system (UPS) is an intracellular proteolytic system mediating the degradation of most intracellular proteins. Studies have shown that the neuroplasticity associated with opioid addiction depends on protein synthesis and degradation, in particular the degradation mediated by the UPS pathway (Caputi et al., 2019; Massaly et al., 2013). Besides, UPS also plays important roles in cocaine‐induced behavioral sensitization (Gonzales et al., 2018) and self‐administration (Werner et al., 2015, 2018). However, the substrate proteins of UPS in drug addiction have not been clarified.

Regulators of G protein signaling (RGS) are a group of regulator proteins of G protein‐coupled receptors. RGS proteins are accelerator proteins of GTPase, which turn off the signal transduction of Gα and βγ of G protein and negatively regulate G protein signaling (McNabb et al., 2020; Senese et al., 2020). So far, there are more than 20 RGS proteins, which are abundantly expressed in many brain regions that mediate addiction and analgesia. Several studies have shown that RGS9‐2, RGS7, and RGS4 participate in regulating drug‐induced plasticity (Gaspari et al., 2014; Kim et al., 2018; Sakloth et al., 2020; Sutton et al., 2016; Wang & Traynor, 2011; Zachariou et al., 2003). In particular, Wang and colleagues have found that treatment of SH‐SY5Y cells with opioid receptor agonists decreased RGS4 protein expression, and the decreased RGS4 protein expression was completely blocked by proteasome inhibitors (Wang & Traynor, 2011). However, the roles of RGS4 and UPS in opioid‐related behaviors have not been investigated.

Taken together, we hypothesize that RGS protein degradation, possibly mediated by UPS, might be related with opioid addiction. In this study, we aimed to conduct a preliminary investigation of the regulatory roles of RGS4 and UPS in the development of morphine‐induced behavioral sensitization in rats, a well‐recognized animal model of opioid addiction (Liu et al., 2021; Trombin et al., 2018).

## 2 MATERIALS AND METHODS

### 2.1 Animals

Male Sprague–Dawley rats initially weighing 240–260 g were obtained from Sibeifu (Beijing) Laboratory Animal Technology Co., Ltd. The animals were housed in transparent plastic cages (*n* = 2–3 per cage) and kept in the light (12 h light/12 h dark, 8:00 a.m. light on), temperature (22 ± 2°C), and relative humidity (50% ± 10%) controlled environment with free access to food and water. The rats were habituated to the housing conditions for at least 5 days and handled carefully for at least 3 days before experiments. The experiments were carried out during the light cycle and conducted in accordance with the NIH Guide for the Care and Use of Laboratory Animals (NIH publications No. 80‐23, revised 1996). The experimental procedures were approved by the local Committee of Animal Care and Use (NCC2018A063).

### 2.2 Drugs

Morphine hydrochloride (morphine, Mor) was purchased from Qinghai Pharmaceutical Manufactory (China). Lactacystin (LAC) was purchased from Tocris. Morphine was dissolved in 0.9% saline and administered subcutaneously (s.c.) at a volume of 0.1 mL/100 g bodyweight. LAC was dissolved in 72.5% dimethyl sulfoxide. Drug doses were adopted as follows: morphine (1, 3, or 10 mg/kg, s.c.) and LAC (400 or 800 ng/rat, microinjection). All the drugs were freshly prepared before the experiments.

### 2.3 Locomotor activity

Rats were put into four identical test chambers (49 cm × 49 cm × 59 cm, without ceiling) and placed in soundproof cabinets immediately after saline/morphine administration, and locomotor activity was measured with a Digbehv spontaneous activity system (Digbehv‐LG; Shanghai Jiliang Software Technology Co. Ltd., Shanghai, China) on both Day 1 and Day 8. Behavioral sensitization was evaluated by the differences of locomotor responses to the challenge morphine on Day 8 between morphine‐ and saline‐pretreated groups with Digbehv software v.2.0.

### 2.4 Cannulae implantation and microinjection

All the surgical instruments, stainless steel guide cannulae, cannulae caps, and screws were sterilized with 75% alcohol in advance. Rats were anesthetized with pentobarbital sodium (70 mg/kg, intraperitoneal injection) and fixed horizontally on a stereotaxic apparatus. Stainless steel guide cannulae (22 gauge) were bilaterally implanted 1 mm above the nucleus accumbens (NAc) core and angled 10° toward the midline. Dental cement was prepared quickly and applied around the catheter and small screws to fix the position of the cannulae. The rats were intraperitoneally injected with penicillin sodium (200,000 units/rat/day) for five consecutive days after the surgery. Each rat was housed in a single cage and was allowed to recover for 5–7 days before further experiments.

Microsyringe, polyethylene (PE) tube, and the internal cannulae were installed before microinjection. The length of the internal cannulae is 1 mm longer than that of the cannulae. Each rat was handled for at least three consecutive days before the microinjection to reduce the postoperative stress response. According to the trajectory of the internal cannulae, the position of the NAc core injection point is calculated as follows: anteroposterior (AP): +1.6 mm; mediolateral (ML): ±1.8 mm; dorsoventral (DV): −7.6 mm (Watson & Paxinos, 2014). LAC was slowly microinjected into the NAc core. The injection volume is 1 μL/side, and the speed is controlled at 0.5 μL/min. After the administration, the internal cannulae were remained in place for another 2 min and pulled slowly to avoid backflow of the drug. At the end of the microinjection, the cannulae cap was screwed again to prevent infection.

At the end of the behavioral tests, all the rats with stereotaxic surgeries were sacrificed, and the coronal slices containing the NAc core were separated. The trajectories of the cannulae placement were compared with the rat brain atlas to confirm that the placements were correct.

### 2.5 Tissue preparation and western blot

Coronal slices containing the NAc core were prepared according to The Rat Brain in Stereotaxic Coordinates (7th edition) (Watson & Paxinos, 2014), and then the NAc core was quickly removed, frozen immediately with liquid nitrogen, and stored at −80°C until processed. The detailed steps for preparation of homogenates and western blot analysis were described previously (Wang et al., 2014).

### 2.6 Experimental design

#### 2.6.1 Experiment 1: The time effect of the single morphine exposure on RGS4 protein expression and polyubiquitination during the development phase of behavioral sensitization (*n* = 4/group)

The rats were treated with normal saline (NS) or 10 mg/kg morphine (s.c.), and the NAc core was removed 2, 4, or 8 h later for testing the target proteins.

#### 2.6.2 Experiment 2: The dose effect of the single morphine exposure on RGS4 protein expression and polyubiquitination during the development phase of behavioral sensitization (*n* = 10/group; the data of one rat were excluded in the analysis of polyubiquitination as the film of western blot was impaired)

The rats were treated with NS or 1, 3, or 10 mg/kg morphine, and the NAc core was removed 2 h later for testing the target proteins.

#### 2.6.3 Experiment 3: The effect of stereotaxic injection of LAC into NAc core on behavioral sensitization induced by a single morphine exposure in rats (*n* = 5–6/group)

All the rats with stereotaxic surgeries were given 5–7 days to recover before the stereotaxic injection of drugs and the behavioral tests. On Day 1, stereotaxic injection of LAC (400 or 800 ng/rat) or vehicle was performed immediately before the systemic administration of morphine (3 mg/kg, s.c.) or NS, and then the locomotive activities of rats were recorded for 180 min. After a 7‐day drug‐free period, all the rats were treated with challenge dose of morphine (3 mg/kg, s.c.), and the locomotive activities of rats were recorded for 180 min.

### 2.7 Statistics

Statistical analysis was performed by IBM SPSS Statistics 19. Data on locomotor activity were analyzed by two‐factor repeated‐measures analysis of variance (ANOVA) with time as a repeated measure (time × treatment). The data of all the column graphs were analyzed by one‐way ANOVA followed by post hoc Tukey's test. All *p*‐values of <.05 were considered as statistically significant.

## 3 RESULTS

### 3.1 The time effect of a single morphine exposure on RGS4 and polyubiquitinated protein expression in the NAc core of rats

Previously, we have successfully established behavioral sensitization induced by a single morphine exposure with the following paradigm: on Day 1, rats were treated with a single dose of morphine (the development phase); on Day 8 (after a 7‐day drug‐free period), all the rats were challenged with a another dose of morphine (could be the same or lower dose compared with the dose on Day 1) (the expression phase) (Qin et al., 2013; Wang et al., 2014).

**FIGURE 1:**
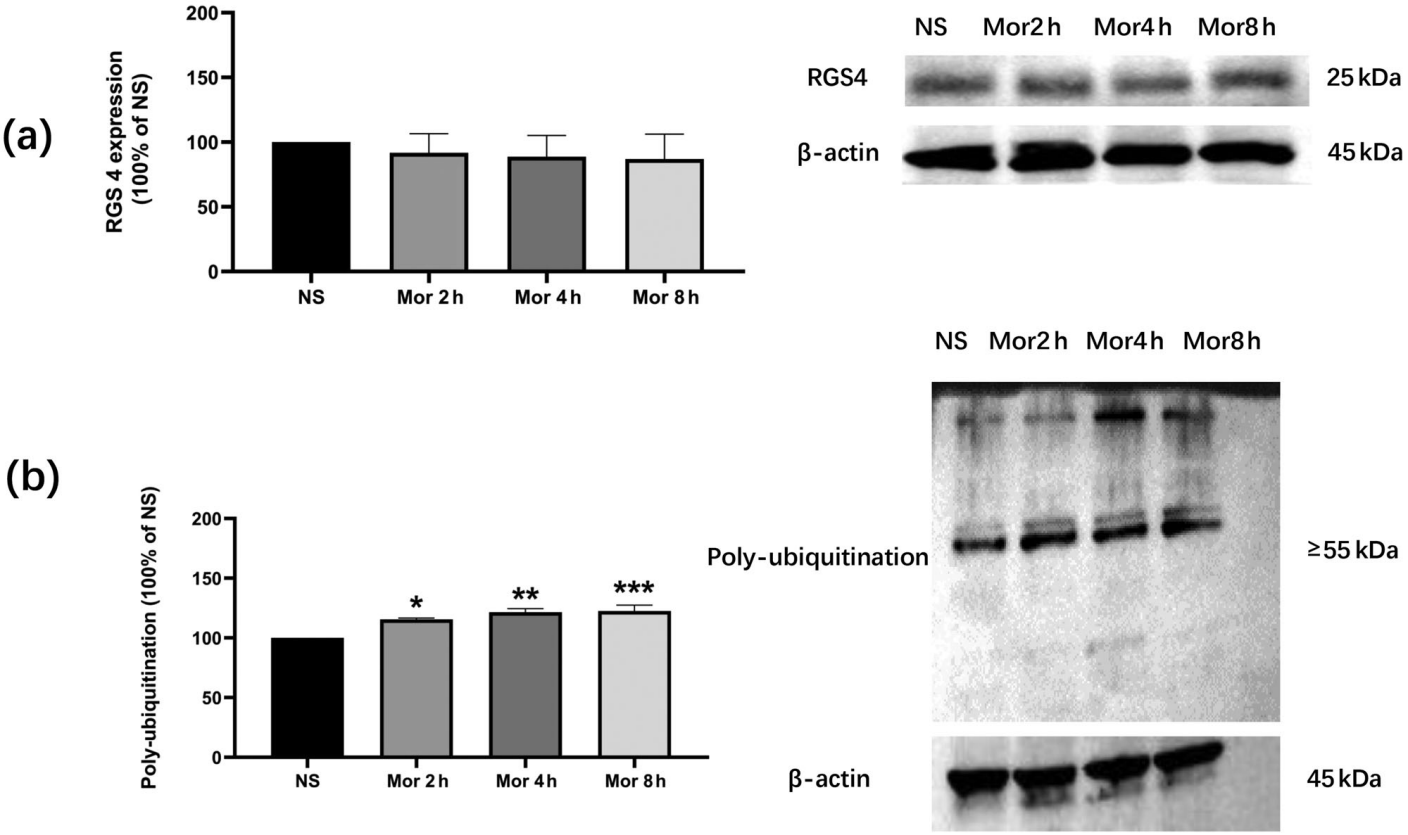
The time effect of a single morphine exposure on the expression of RGS4 and polyubiquitination in NAc core of rats. (a) Time effect of a single morphine injection on RGS4 expression in NAc core of rats. RGS4 expression was not significantly changed at the time point of 2, 4, and 8 h (*n* = 4/group). (b) Time effect of a single morphine injection on polyubiquitination expression in NAc core of rats. Polyubiquitination expression was significantly increased at the time point of 2, 4, and 8 h (*n* = 4/group). All data are expressed as means ± SEM. *p < .05, **p < .01, and ***p < .001 versus NS group.

To explore the changes of our target proteins during the development phase of behavioral sensitization, we first investigated the time effect of the single morphine exposure on RGS4 and polyubiquitinated protein expression. The rats were treated with saline or 10 mg/kg morphine, and the NAc core was removed 2, 4, or 8 h later for testing the target proteins. RGS4 expression was not significantly changed at the time points of 2, 4, and 8 h (one‐way ANOVA: *F*_(3, 12)_ = 0.1536, *n* = 4/group, *p* > .05; Tukey's multiple comparisons test: Mor 2 h vs. NS: *p* > .05; Mor 4 h vs. NS: *p* > .05; Mor 8 h vs. NS: *p* > .05) (Figure 1a). However, polyubiquitinated protein expression was significantly increased at 2, 4, and 8 h (one‐way ANOVA: *F*_(3, 12)_ = 12.08, *n* = 4/group, *p* < .001; Tukey's multiple comparisons test: Mor 2 h vs. NS: *p* < .05; Mor 4 h vs. NS: *p* < .01; Mor 8 h vs. NS: *p* < .001) (Figure 1b).

### 3.2 The dose effect of a single morphine exposure on RGS4 and polyubiquitinated protein expression in the NAc core of rats

**FIGURE 2:**
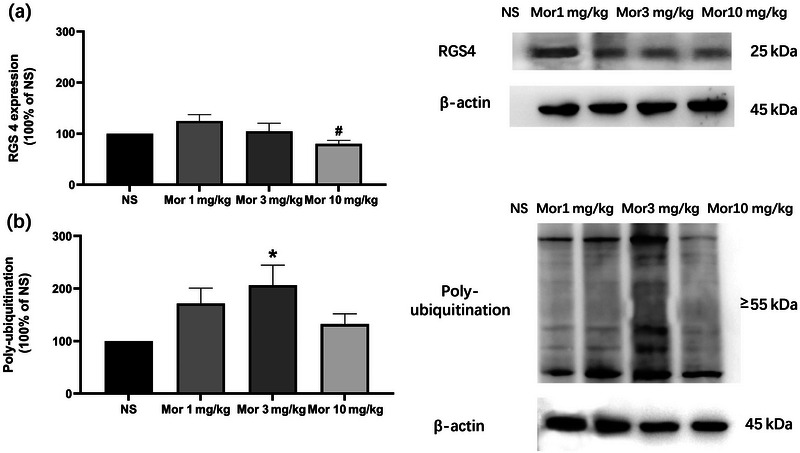
The dose effect of a single morphine exposure on RGS4 and polyubiquitination in NAc core of rats. (a) Dose effect of a single morphine injection on RGS4 expression in NAc core of rats. RGS4 expression was not changed in morphine groups when being compared with NS group (*n* = 10/group). (b) Dose effect of a single morphine injection on polyubiquitination expression in NAc core of rats. Polyubiquitination expression tended to be increased in Mor 1 mg/kg group, with 3 mg/kg group showing significant difference compared to NS group (*n* = 9/group). All data are expressed as means ± SEM. *p < .05 versus NS group; #*p* < .05 versus Mor 1 mg/kg group.

The rats were treated with saline or 1, 3, or 10 mg/kg morphine, and the NAc core was removed 2 h later for testing the target proteins. RGS4 expression was not changed in morphine groups when being compared to NS group (one‐way ANOVA: *F*_(3, 36)_ = 2.964, *n* = 10/group, *p* < .05; Tukey's multiple comparisons test: Mor 1 mg/kg vs. NS: *p* > .05; Mor 3 mg/kg vs. NS: *p* > .05; Mor 10 mg/kg vs. NS: *p* > .05; Mor 10 mg/kg vs. Mor 1 mg/kg: *p* < .05) (Figure 2a). Additionally, polyubiquitinated protein expression tended to be increased in Mor 1 mg/kg group, with Mor 3 mg/kg group showing significant difference compared to NS group (one‐way ANOVA: *F*_(3, 32)_ = 3.152, *n* = 9/group, *p* < .05; Tukey's multiple comparisons test: Mor 1 mg/kg vs. NS: *p* > .05; Mor 3 mg/kg vs. NS: *p* < .05; Mor 10 mg/kg vs. NS: *p* > .05) (Figure 2b).

### 3.3 The effect of stereotaxic injection of LAC into NAc core on behavioral sensitization induced by a single morphine exposure in rats

**FIGURE 3:**
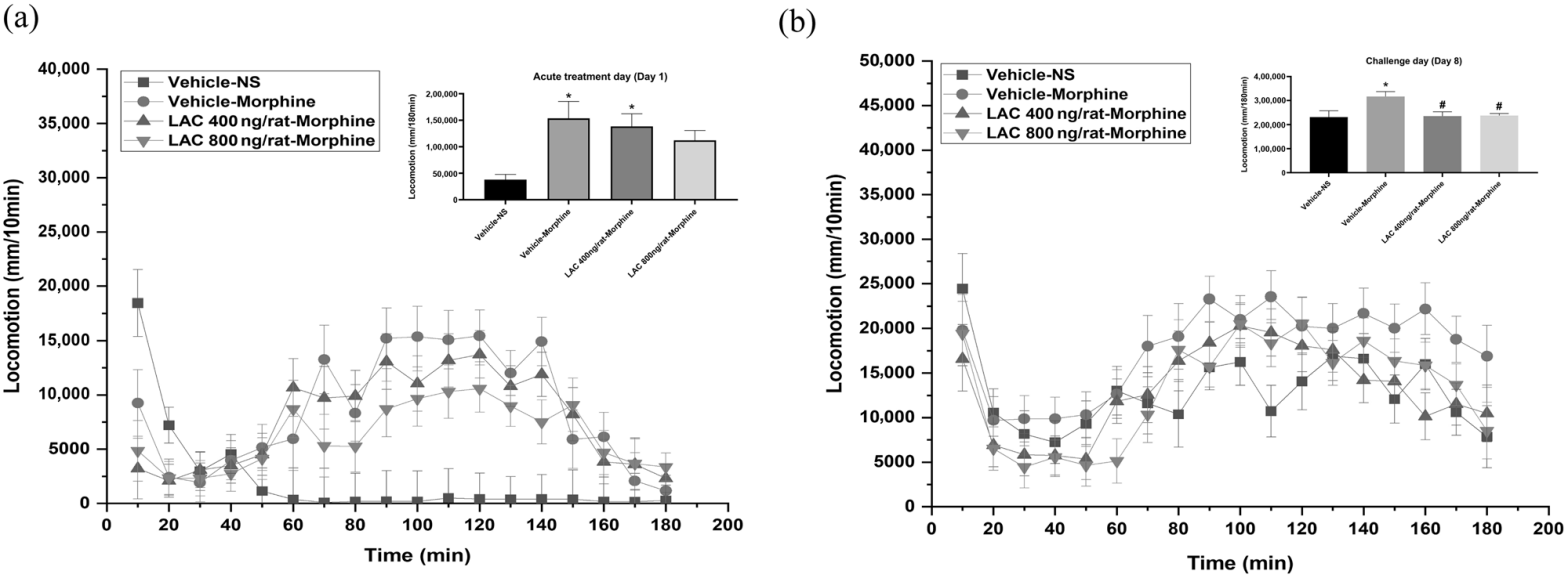
The effect of stereotaxic injection of LAC in NAc core on behavioral sensitization induced by a single morphine exposure in rats. (a) The effect of stereotaxic injection of LAC in NAc core on the hyperlocomotion induced by a single morphine exposure in rats. The single morphine exposure could induce hyperlocomotion. LAC at neither dose of 400 nor 800 ng/rat did have an effect on acute morphine‐induced hyperlocomotion (*n* = 5–6/group). (b) The effect of stereotaxic injection of LAC in NAc core on behavioral sensitization. Behavioral sensitization was successfully established. LAC at both doses of 400 and 800 ng/rat impaired behavioral sensitization (*n* = 5–6/group). The name of each group represents different treatments on Day 1. All data are expressed as means ± SEM. *p < .05 versus Vehicle–NS group; #*p* < .05 versus Vehicle–Morphine group.

On Day 1, stereotaxic injection of LAC/Vehicle was administered immediately before the systemic administration of morphine/saline. The single morphine exposure could induce hyperlocomotion (*F*_(3, 18)_ = 4.839, *p* < .05, *n* = 5–6/group; Tukey's test: Vehicle–Morphine vs. Vehicle–NS: *p* < .05). The time effect (“time” represents every 10 min in the locomotor chamber) of stereotaxic injection of LAC on locomotor activity was also investigated (*F*_(time)17,306_ = 7.804, *p* < .001; *F*_(treatment)3, 18_ = 4.839, *p* < .05; *F*_(time × treatment)51,306_ = 3.362, *p* < .001) (Figure 3a). LAC at doses of 400 and 800 ng/rat did not have significant effects on acute morphine‐induced hyperlocomotion (*p* > .05 vs. Vehicle–Morphine group) (Figure 3a, insect).

On Day 8, the time effect of LAC on behavioral sensitization was also investigated (*F*_(time)17, 306_ = 11.256, *p* < .001; *F*_(treatment)3,18_ = 4.558, *p* < .05; *F*_(time × treatment)51,306_ = 0.896, *p* > .05) (Figure 3b). In the inset of Figure 3b, the total locomotor activity was further analyzed by one‐way ANOVA (*F*_(3, 18)_ = 4.558, *n* = 5–6/group, *p* < .05) and post hoc Tukey's test. The locomotion of Vehicle–Morphine group was higher than that of the Vehicle–NS group, suggesting the establishment of behavioral sensitization (*p* < .05 vs. Vehicle–NS group). LAC at both doses of 400 and 800 ng/rat impaired the establishment of behavioral sensitization (*p* < .05 vs. Vehicle–Morphine group).

## 4 DISCUSSION

This study explored the involvement of UPS and RGS4 in behavioral sensitization, a well‐recognized animal model of opioid addiction. First, polyubiquitination expression in the NAc core was time dependently and dose relatedly increased during the development of behavioral sensitization, while RGS4 protein expression was not significantly changed during this time period. Second, stereotaxic administration of LAC into the NAc core could impair the establishment of behavioral sensitization.

The proteasome is a key element of the UPS, which is responsible for over 80% of cellular protein degradation (Omura & Crump, 2019). In the present study, we explored the effect of a selective proteasome inhibitor, LAC, on the establishment of behavioral sensitization. We found that LAC, locally given into NAc core, could impair behavioral sensitization. LAC is the first natural nonpeptidic proteasome inhibitor, which potently and irreversibly inhibits specific catalytic subunits of the proteasome (Fenteany et al., 1995), and is widely considered as the primary reagent for studying the role of the proteasome (Omura & Crump, 2019). In this study, the effect of LAC on the establishment of behavioral sensitization indicates that UPS is positively involved in behavioral sensitization induced by a single morphine exposure. Besides, several lines of evidence reported the roles of UPS in molecular and cellular consequences of the treatment of different kinds of drugs of abuse (Massaly et al., 2014), including stimulants (Guan & Guan, 2013; Mao et al., 2009; Ren et al., 2013), ethanol (Gutala et al., 2004; Pla et al., 2014), and nicotine (Henley et al., 2013; Kane et al., 2004; Rezvani et al., 2007). Similarly, at the behavioral level, UPS plays a vital role in cocaine‐induced behavioral sensitization (Gonzales et al., 2018) and self‐administration (Werner et al., 2015, 2018). The above results together indicate that UPS plays a positive regulatory role in drug addiction.

To explore the possible substrate of UPS, we also measured the expression of RGS4 in the development of behavioral sensitization. Based on our results, the expression of RGS4 in NAc core was not significantly changed during the development of behavioral sensitization. However, at the cell level, Wang and colleagues (Wang & Traynor, 2011) found that there was a downregulation of RGS4 protein expression induced by opioid receptor agonists, which could be reversed by proteasome inhibitors. Back to our animal study, we found that RGS4 protein expression was not downregulated during the development of behavioral sensitization. The reasons for the difference between the two studies may be as follows: (1) the experimental subjects were different. Wang and colleagues conducted the research at the cellular level, and measured RGS4 expression in human neuroblastoma SH‐SY5Y cells; however, our study is at the animal level and we measured RGS4 expression in NAc core of rats. (2) The drugs were different. Wang and colleagues used mu‐opioid agonist DAMGO and delta‐opioid receptor agonist DPDPE, and we used mu‐opioid receptor agonist morphine. (3) The durations of drug treatment were different. The cells in Wang paper were treated overnight with DAMGO or DPDPE; however, the rats in our study were exposed for only one injection of morphine. Hence, it is likely that other family members of RGS proteins or even other proteins might act as substrate proteins for UPS‐dependent protein degradation in behavioral sensitization. For example, several lines of evidence have shown that RGS9‐2 and RGS7 have unique mechanisms in regulating drug‐induced plasticity, and they are both negative regulators of opioid‐induced addictive behaviors (Gaspari et al., 2014; Sutton et al., 2016; Zachariou et al., 2003). Additionally, synaptic anchoring proteins (Lee et al., 2008) and transcription factors (Carle et al., 2007; Dong et al., 2008) might also be direct or indirect targets of UPS. Therefore, these could be the direction for our future exploration of the roles of the substrate proteins of UPS in opioid addiction.

## 5 CONCLUSION

UPS in NAc core is positively involved in behavioral sensitization induced by a single morphine exposure in rats. Polyubiquitination was observed during the development phase of behavioral sensitization, while RGS4 protein expression was not significantly changed, indicating that other members of RGS family might be substrate proteins in UPS‐mediated opioid addiction.

## CONFLICT OF INTEREST STATEMENT

The authors declare no conflicts of interest.

### PEER REVIEW

The peer review history for this article is available at https://publons.com/publon/10.1002/brb3.2922

## Data Availability

The data that support the findings of this study are available from the corresponding author upon reasonable request.
